# An Internal Reference Control Duplex Real-Time Polymerase Chain Reaction Assay for Detecting Bacterial Contamination in Blood Products

**DOI:** 10.1371/journal.pone.0134743

**Published:** 2015-07-31

**Authors:** Jin-ju Zhang, Jing-jing Tian, Shuang-shi Wei, Sheng-bao Duan, Hong-mei Wang, Ye-zhou Chen, Shao-hua Ding, Chun Zhang, Qing-lin Meng, Yong Li

**Affiliations:** The Chinese Academy of Sciences Key Laboratory of Bio-Medical Diagnostics, Suzhou Institute of Biomedical Engineering and Technology, Chinese Academy of Sciences, Suzhou, Jiangsu, China; Robert Koch-Institute, GERMANY

## Abstract

Real-time polymerase chain reaction (RT-PCR) enables effective and sensitive screening for infectious risk in the field of blood safety. However, when using RT-PCR to detect bacterial contamination, several intractable points must be considered, one of which is the lack of appropriate quality control. In this study, we developed a simplified RT-PCR assay in which the same primer set and two distinct probes were used to detect both, an internal reference control and the target in a reaction. The copy number of the internal reference control represents the positive detection limit of the assay; therefore, when the threshold-cycle value of the target is less than or equal to that of the internal reference control, the result obtained for the target can be considered to be a true positive. When human gDNA was spiked with *Escherichia coli* gDNA and the detection limit for the internal reference control was set to five copies, the measured detection limit for *E*. *coli* gDNA was two copies. The internal reference control duplex RT-PCR assay showed high efficiency (0.91–1.02), high linearity (*R^2^* > 0.99), and good reproducibility in intra- and inter-assay comparisons. Lastly, when human platelet-rich plasma samples were spiked with *E*. *coli* or other bacterial species, all species were detected efficiently, and the results of a two-sample pooled t test showed that the limit of detection for E. coli was 1 cfu/mL. Here, we present a synthetic internal reference control molecule and a new statistical method for improving the reliability of RT-PCR assays when screening for bacterial contamination in blood products.

## Introduction

The transmission of viral diseases during transfusion has declined substantially during the past two decades, but septic transfusion reactions resulting from bacterial infection have emerged as the major cause of transfusion-related morbidity and mortality [1]. Among blood-derived products, platelet concentrates (PCs) are particularly prone to bacterial contamination because the conditions used for preserving PC function and survival (22 ± 2°C and constant agitation) favor bacterial growth. Based on a literature review, the contamination level was previously estimated to be 1 in 2,000–3,000 units of PCs and the risk for severe septic infection caused by platelet transfusion was estimated to range from 1:50,000 to 1:150,000 [2]. Consequently, in 2004, the American Association of Blood Banks published standards for detecting and diminishing bacterial contamination in all platelet components [3].

The classical BacT/ALERT assay system (bioMérieux Inc.) developed for bacterial-contamination screening, which is currently widely used, is based on measuring carbon dioxide production [4]. This system, together with the Pall eBDS system, has been approved by the US Food and Drug Administration and is now used at transfusion centers for routine screening of PCs. The Platelet PGD Test (Verax Biomedical, Inc.) is a new quantitative immunoassay that is used in addition to the aforementioned assay systems; this test can be rapidly applied and is the preferred method for secondary screening or for procedures that involve the emergency release of PCs [5].

The current culture methods used for PC screening can be time-consuming and expensive, and when using these methods, the slow growth of microorganisms coupled with very low bacterial loads is likely to generate false-negative results [6]. Given these limitations, alternative or supplementary methods have been developed to improve PC screening. Previous studies have reported several nucleic acid-testing assays that target 16S/23S ribosomal DNA/RNA for the detection of a broad range of bacterial species by using real-time polymerase chain reaction (RT-PCR) [7–9]. RT-PCR is an appealing alternative to conventional culture methods because the assay theoretically allows precise quantification of a specific sequence of nucleic acid in a complex mixture even if the starting amount of material is at a very low concentration. Nevertheless, in the practical implementation of RT-PCR, one of the major challenges is the ability to distinguish with confidence the presence of false-positive or false-negative results [10]. This reliability concern has been addressed by using multiplex RT-PCR assays; in these assays, extra primer-probe sets that target endogenous or exogenous internal controls are added. However, the detection efficiency of multiplex RT-PCR can be diminished because of differences in sequence and secondary-structure for extra primers and probes, and because multiple competitive reactions can occur [11]. Furthermore, for determining copy numbers of target, external copy-number standard curves are required, and the methods of obtaining these pose an additional risk of contamination.

In this study, we developed a simplified format for an RT-PCR assay; the assay includes a specified number of internal reference control (IRC) template molecules, target probes, IRC probes labeled with distinct fluorophores, and a single pair of primers for amplifying both the target gene and the IRC. In this assay format, the IRC copy number represents the limit of positive detection and it is set as the internal control. Because the threshold-cycle value and the template copy number are negatively correlated, when the threshold-cycle value of the target (Cts) is less than or equal to that of the IRC (Cti), the detection result for the target can be considered a true positive. When the difference between Cts and Cti is statistically significant, the result can be readily interpreted as being a true-positive or -negative result; however, when the difference is not statistically significant, a two-sample pooled *t* test is used to determine whether Cts ≤Cti. Therefore, our new system offers an IRC to minimize false-negative results and establish true-positive results.

## Materials and Methods

### Ethics statement

Human whole-blood samples were obtained from healthy donors at our institute in accordance with the standard operating protocols of the National Guide to Clinical Laboratory Procedures (Third Edition, 2006) that are approved by the Department of Medical Administration, Ministry of Health, and People’s Republic of China. Written informed consent was obtained from participants before enrollment. The local ethics committee of Suzhou Institute of Biomedical Engineering and Technology, Chinese Academy of Sciences, reviewed and approved all procedures used in this study (SIBET-JL701-JYC04-2014).

### Bacterial strains and spiking of platelet-rich plasma

The bacterial strains used in this study were *Bacillus cereus* (isolate DWL062), *Escherichia coli* (ATCC 44155), *Enterobacter cloacae* (isolate HHHT M0801), *Pseudomonas aeruginosa* (ATCC 27853, isolate DGY2722), *Propionibacterium acnes* (isolate jj0401), *Staphylococcus aureus* (ATCC 25923, isolate jj0402), and *S*. *epidermidis* (ATCC 12228); the strains were cultured in Nutrient Broth medium at 37°C. The genomic copy number was calculated based on DNA concentrations and bacterial genome sizes. To obtain bacterial titers, overnight cultures were diluted in fresh medium to a density of 0.5 McFarland standard units (approximately 1.5 × 10^8^ cfu/mL) and then 10-fold serially diluted (10^−4^ to 10^−7^); subsequently, 100-μL aliquots of the serial dilutions were plated on Nutrient Broth agar plates and the single colonies that formed were counted. Cultures of *E*. *coli* and other bacterial species in the exponential growth phase were 10-fold serially diluted (10^−4^ to 10^−8^); subsequently, 100-μL aliquots of these serial dilutions were spiked into 1 mL of platelet-rich plasma for use in DNA coextraction assays.

### Nucleic acid extraction

Human gDNA samples were extracted from 0.5–2 mL of human whole-blood samples by using the TIANamp Blood DNA Midi Kit (DP332; Tiangen Biotech, Beijing, China) and eluted in 50 μL. The total DNA was isolated from 2 mL of the platelet-rich plasma spiked with bacterial species by using the same kit and eluted in 50 μL; bacterial gDNA was purified using the TIANamp Bacteria DNA Kit (DP302; Tiangen Biotech) and eluted in 50 μL. All procedures were performed in accordance with the manufacturer’s instructions. The concentration and purity of the isolated nucleic acids were determined using a NanoDrop 2000 spectrophotometer.

### Primer and probe design

The universal primers and probes used for detecting bacterial 16S/23S rRNA were designed using Vector NTI software (Life Technologies, Foster City, CA, USA), based on the sequences of the *E*. *coli* K12-related 16S rRNA gene (NCBI Gene ID: 944897) and 23S rRNA gene (NCBI Gene ID: 944900). The primer and probe sequences were screened against the human genome in the UCSC In-Silico PCR database (http://genome.ucsc.edu/cgi-bin/hgPcr?command=start); Table 1 lists the primers and probes used in this study. All oligonucleotide TaqMan probes were synthesized and HPLC purified (>98%) by Shanghai Sangon (Shanghai, China).

**Table 1:** Primers and probes used in this study.

Oligonucleotide	Sequence (5’-3’)
16S-F (897–914)	CGCAAGGYTRAAACTCAA
16S-R (1066–1083)	ATYTCACRACACGAGCTG
16S-FAM-probe (959–977)	FAM-ATTCGAHGCAACGCGWAGA-BHQ1
23S-F (2438–2455)	TACYCYGGGGATAACAGG
23S-R (2591–2608)	CCGAACTGTCTCACGACG
23S-FAM-probe (2492–2509)	FAM-TTGGCACCTCGATGTCGG-BHQ1
IRC-CY3-probe	CY3-CCTCACTCGAACTGGCCG-BHQ2

F = forward; R = reverse; Y = C/T; R = A/G; H = A/T/C; W = A/T; BHQ1 = dark quencher 480–580 nm; BHQ2 = dark quencher 520–650 nm

### IRC design

When designing an appropriate artificial IRC template, several factors must be considered, including the following: (1) the target probe and the IRC probe must have a similar Tm; (2) both amplification products must have a similar Tm; (3) no homology should exist between the target probe and the IRC template and between the IRC probe and the target sequences; and (4) the secondary structure of the IRC must not be intricate. We designed the IRC templates based on these factors, and the templates included sequences corresponding to the 16S/23S forward and reverse primers at 5' and 3' termini and an artificial DNA fragment containing a specific probe-binding site. The IRC templates were generated by annealing two complementary synthetic single-stranded oligonucleotides. The sequence of the forward 16S IRC strand was 5'-CGCAAGGTTGAAACTCAACCAGCTGCCAGTCCTCACTCGAACTGGCCGTC TACAGTCTTCCAGGACAGCTCGTGTCGTGAGAT-3', and the complementary sequence was 5'-ATCTCACGACACGAGCTGTCCTGGAAGACTGTAGACG GCCAGTTCGAGTGAGGACTGGCAGCTGGTTGAGTTTCAACCTTGCG-3'. The internal sequence of the 23S IRC molecule was identical to that of the 16S IRC, whereas the 5' and 3' flanking sequences were replaced with the 23S forward and reverse primer sites, respectively. The sequence of the forward 23S IRC strand was 5'-TACTCCGGGGATAACAGGCCAGCTGCCAGTCCTCACTCGAACTGGCCGTC TACAGTCTTCCAGGACGTCGTGAGACAGTTCGG-3' and the complementary sequence was 5'-CCGAACTGTCTCACGACGTCCTGGAAGACTGTAGACGG CCAGTTCGAGTGAGGACTGGCAGCTGGCCTGTTATCCC CGGAGTA-3'.

### RT-PCR program

Before performing the RT-PCR assay, the master mix without any added sample DNA was digested for 20 min at 65°C with 1 U of *Taq* ^α^I (R0149V; NEB, Beijing, China) in order to decontaminate the PCR reagent; subsequently, the enzyme was heat-inactivated at 80°C for 20 min. RT-PCR assays (final volume, 50 μL) were performed using the Premix Ex Taq qPCR Kit (RR390A; TaKaRa, Dalian, China) and an ABI 7500 instrument (Applied Biosystems, Foster City, CA, USA). For the probes and primers, concentrations of 250 nM and 800 nM were found to be optimal, respectively, and DMSO was added to a final concentration of 7.5% in order to increase sensitivity. The PCR cycling program was as follows: 50°C for 2 min, 95°C for 10 min, and then 40 cycles of 95°C for 15 s and 60°C for 1 min. Four replicates were used in each experiment.

### Calculation of the RT-PCR limit of detection

For determining the limit of detection, 10-fold serial dilutions of the DNA template were prepared (from 1,000 copies to 1 copy) and amplified using the optimized conditions. Negative controls were included in each experiment, four replicates were used for each reaction, and the minimum positive concentrations in the standard curve (*R* ^*2*^ > 0.99) were considered as the limits of detection. The limit of detection of the IRC was set as the minimum copy number of the positive internal control; thus, the limit of detection of the 16S/23S target was determined based on both serial titration and the hypothesis that Ct_16s/23S_ ≤ Ct_IRC_.

### Statistical analysis

The raw fluorescence emission data were processed using ABI 7500 software to obtain Ct values, linearity (*R* ^*2*^), and efficiency. Microsoft Excel software was used for performing Student’s *t* tests and other statistical analyses. A two-sample pooled variance *t* test was employed to detect the true difference between two groups featuring small sample sizes. The following hypothesis was formulated: *H*_*0*_: Cts ≤ Cti; *H*_*A*_: Cts > Cti; confidence level (α): 10%; if *t*_*c*_ > *t*_*(α*, *2n-2)*_, then reject *H*_*0*_ at α = 0.10.

$$t_{c}\;=\;\frac{\bar{C_{ts}}\;-\;\bar{C_{ti}}}{S_{pooled}}\;\text{where }S_{pooled}\;=\;\sqrt{\frac{\left(n_{1}-1\right)S_{1}^{2}\;+\;\left(n_{2}-1\right)S_{2}^{2}}{n_{1}\;+\;n_{2}\;-\;2}}\text{ *}\sqrt{\frac{n_{1}+\;n_{2}}{n_{1}\text{ * }n_{2}}}$$

where *n* is the number of replicates and *S*_*1*_ and *S*_*2*_ are the standard deviations of Cts and Cti, respectively.

## Results

### IRC duplex RT-PCR format

**Fig 1:**
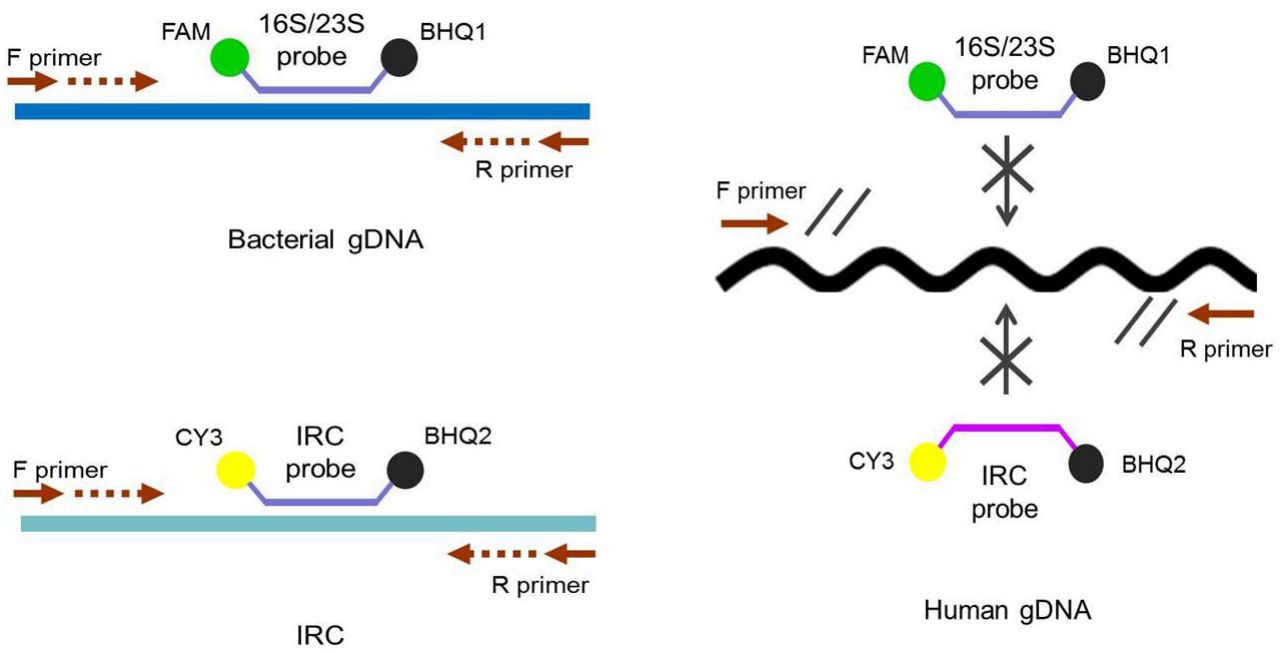
Internal reference control (IRC) duplex RT-PCR assay format. The target and IRC templates share the same primer set, the specific probes for the target and IRC are labeled with different fluorescent dyes, and the assay is performed in one reaction.

**Fig 2:**
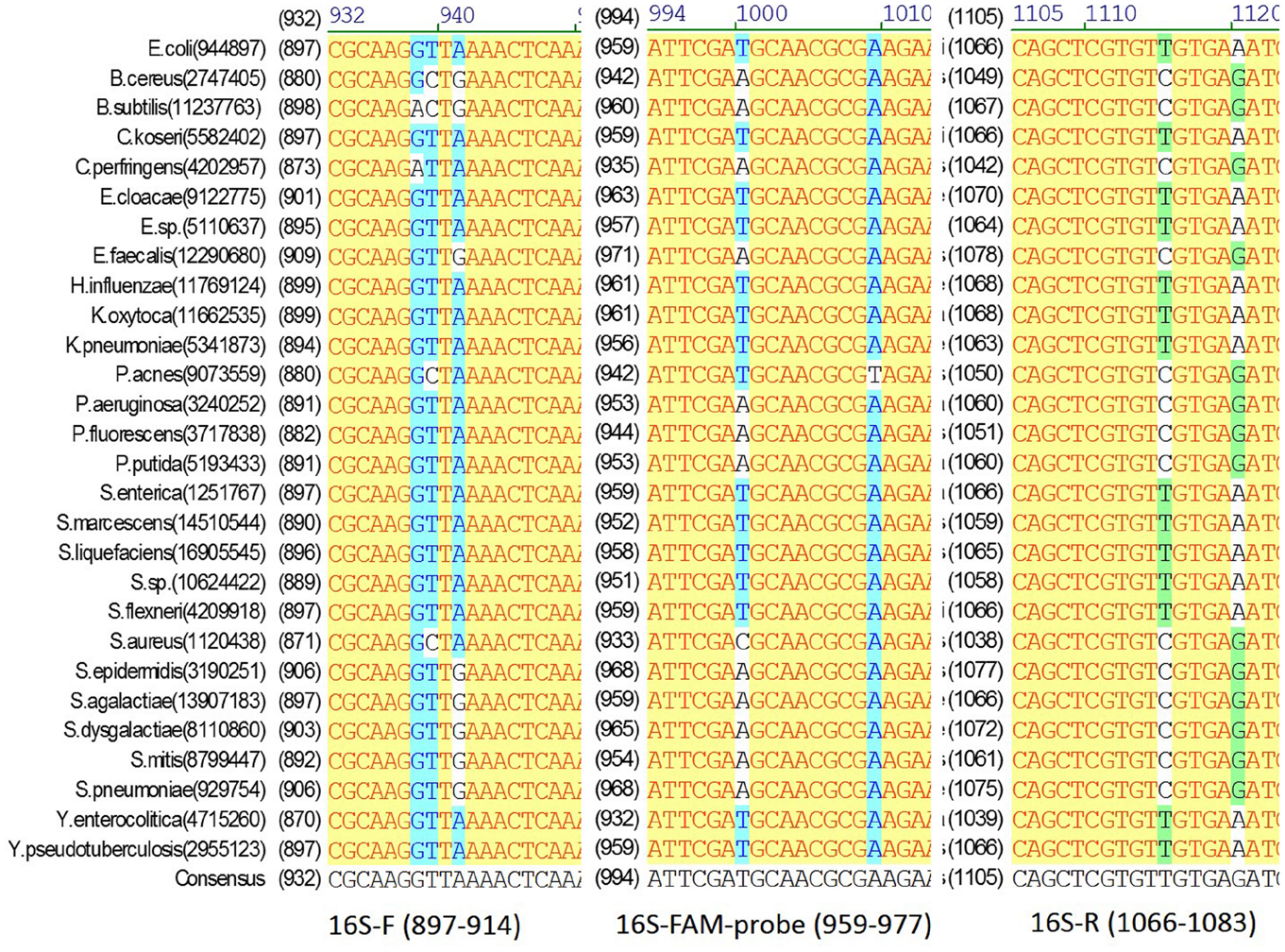
Multiple sequence alignment of the universal 16S primers and probes with the 16S rDNA sequences. The 16S rDNA sequences were obtained from 28 bacterial species (their names and NCBI Gene IDs are shown on the left of the alignment).

**Fig 3:**
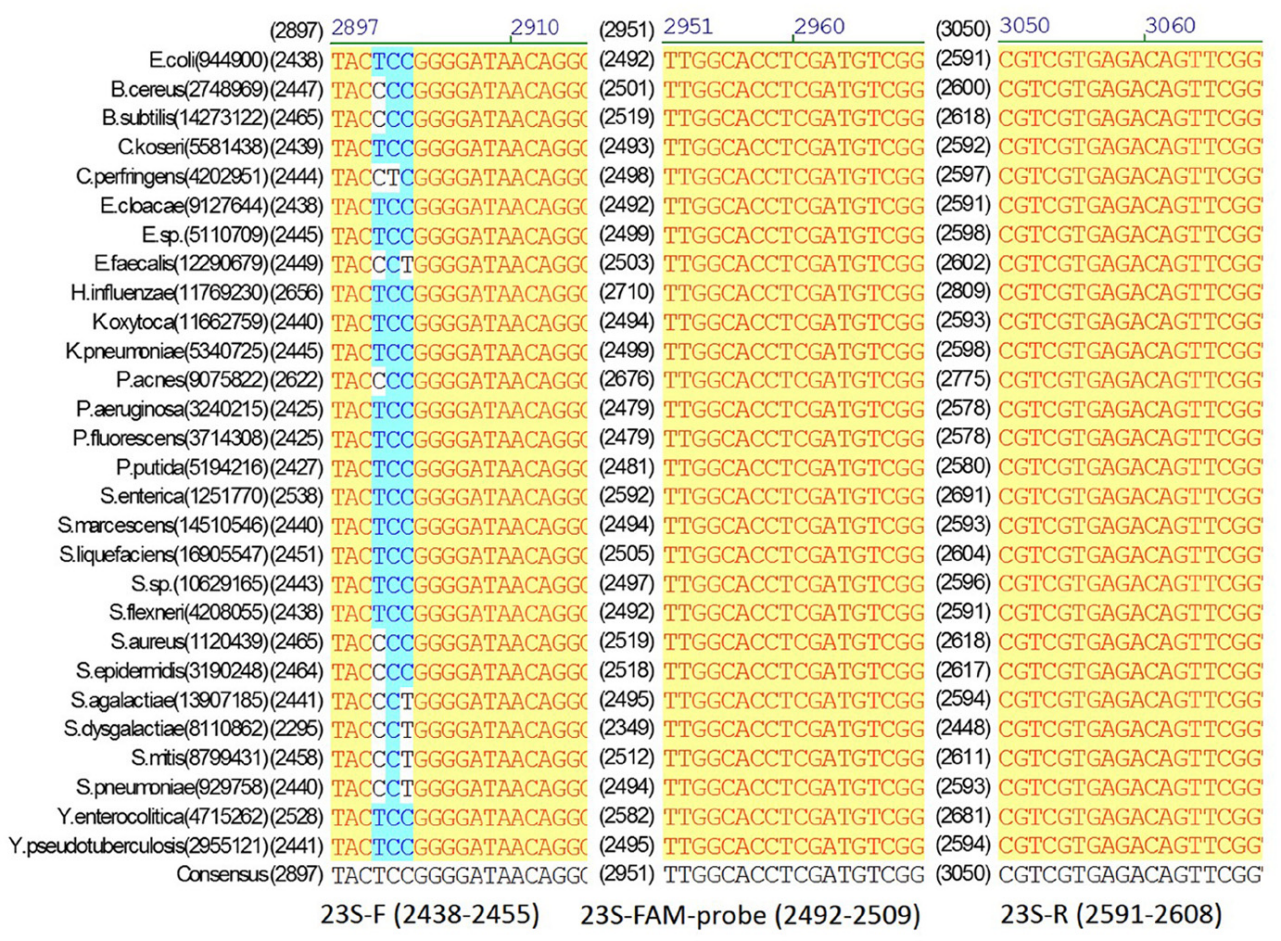
Multiple sequence alignment of the universal 23S primers and probes with the 23S rDNA sequences. The 23S rDNA sequences were obtained from 28 bacterial species (their names and NCBI Gene IDs are shown on the left of the alignment).

**Fig 4:**
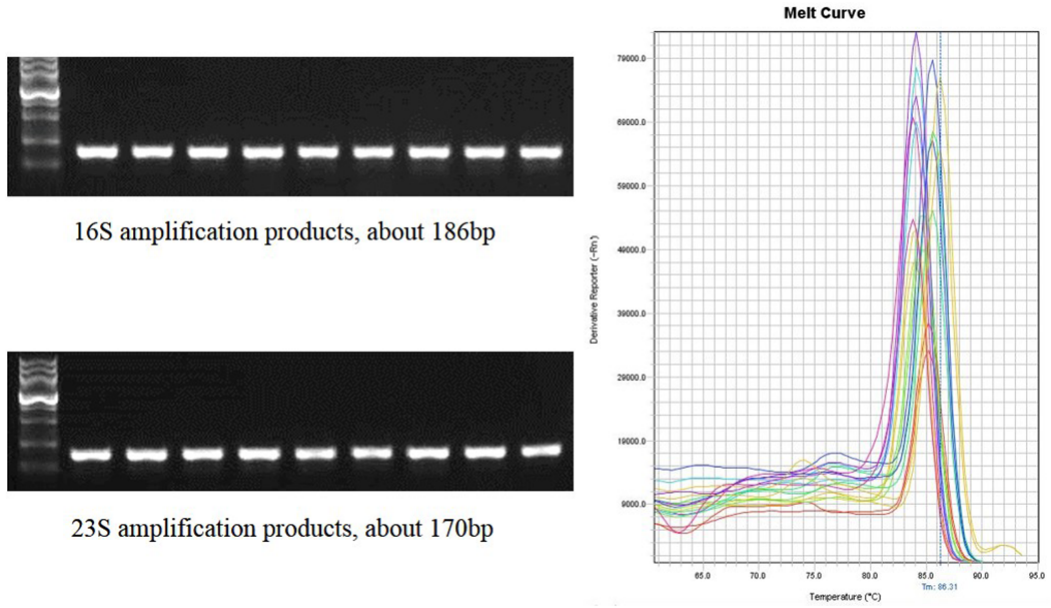
Specificity testing of the 16S/23S primers. PCR and melt curve analyses were used to detect nine bacterial strains and evaluate the specificity of the 16S/23S primers.

**Fig 5:**
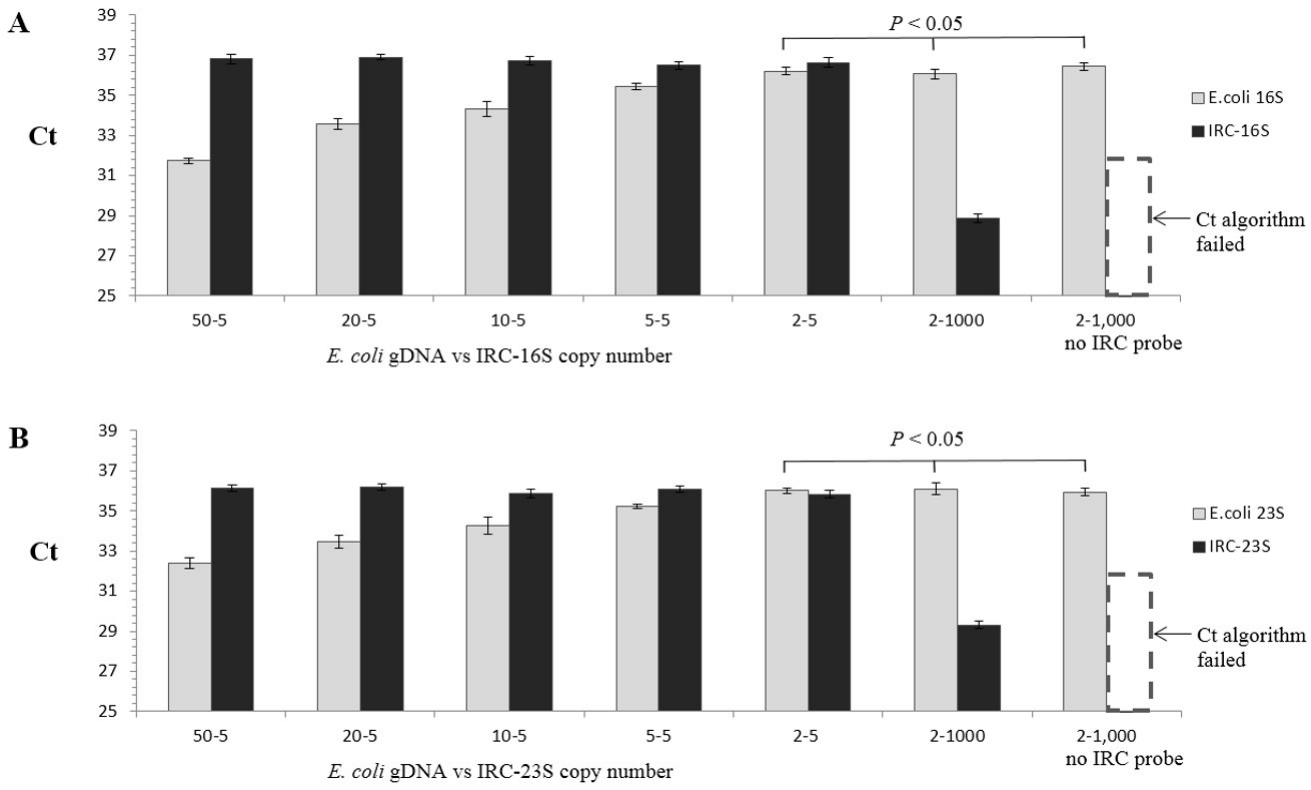
Internal reference control (IRC) duplex RT-PCR: testing for reliability, competition, and inhibition. The copy number of the IRC template was held at five copies, whereas the *E*. *coli* gDNA was titrated from two copies to 50 copies. A: IRC-16S duplex RT-PCR assay. B: IRC-23S duplex RT-PCR assay.

In the RT-PCR assay format developed in this study, an IRC template and a corresponding IRC probe was used (Fig 1). The primers used for amplifying 16S/23S rRNA target genes can be redesigned and used for a wide range of bacterial species. To validate the specificity of the primers (Figs 2 and 3), the 16S/23S rDNA sequences of 28 bacterial species associated with PC contamination [7,12] were selected to generate a multiple sequence alignment by using AlignX (Vector NTI Suite, Life Tech.). Several bacterial species, including standard strains and clinical isolates, were used for testing the specificity of the two pairs of primers for 16S/23S rDNA, and the results of the melting curve analysis showed that neither nonspecific products nor primer dimers were present (Fig 4). Consistent with analyses conducted using NCBI BLAST and the UCSC In-Silico PCR database, no cross-hybridization was observed between primers, primers and probes, and primer/probe sets with human-genome plus transcripts. The results of inhibition testing demonstrated that the presence or absence of the IRC probe had no effect on the 16S/23S probe in the PCR assays (Fig 5).

In our IRC assay format, specific recognition sites (TCGA) were included at the probe-binding region in the target 16S/23S rRNA gene fragment to allow *Taq* ^α^I to digest possible residual templates before testing.

### Performance of the IRC duplex RT-PCR assay

First, we performed RT-PCR assays and plotted standard curves in order to determine the IRC detection limit, which was measured to be five copies under optimized conditions. Subsequently, we performed the IRC duplex RT-PCR assays in which five copies of the IRC were added to each reaction, and we prepared serial dilutions of *E*. *coli* gDNA and detected the gDNA under the same conditions. The results showed that two copies of *E*. *coli* gDNA could be detected using this assay (Table 2). The results of the IRC duplex RT-PCR assays further showed that the assay was highly efficient (0.91–1.02) and highly linear, with *R* ^*2*^ being >0.99 both in the presence and the absence of 500 ng of human gDNA (Table 2).

**Table 2:** Sensitivity, linearity, and efficiency of internal reference control (IRC) duplex RT-PCR.

Assay	In absence of 500 ng human gDNA			In presence of 500 ng human gDNA		
	LOD/mean Cts	*R* ^*2*^	Eff. %	LOD/mean Cti	*R* ^*2*^	Eff. %
IRC^16S^	5 copies36.19 ± 0.15	0.997	101.3	5 copies36.07 ± 0.35	0.992	100.7
16S	2 copies gDNA35.47 ± 0.29	0.991	91.5	2 copies gDNA35.76 ± 0.29	0.995	96.2
IRC^23S^	5 copies35.56 ± 0.13*	0.992	100.3	5 copies36.28 ± 0.35	0.992	97.7
23S	2 copies gDNA35.73 ± 0.22*	0.993	94.6	2 copies gDNA35.72 ± 0.31	0.991	102.1

* The two-sample pooled *t*-test analysis showed that Cts of 23S ≤ Cti of IRC^23S^.

In the IRC duplex RT-PCR, the amplicons of both the target and the IRC share the same pair of primers; thus, we analyzed the possibility of primer competition or mutual inhibition. Our results showed that the amplification of *E*. *coli* gDNA was not affected by the presence or absence of the IRC (Fig 5). Furthermore, when the IRC copy number was fixed at five and the *E*. *coli* gDNA was titrated from 2 to 50 copies, the calculated Ct of the IRC was stable, but that of the target increased when the *E*. *coli* gDNA copy number was decreased (Fig 5); a similar pattern of results was observed in all assays. These results indicated a lack of primer interference or competition and reflected the reproducibility of the IRC duplex RT-PCR assay in intra- and inter-assay comparisons.

### Testing platelet-rich plasma spiked with *E*. *coli* and other bacterial species

Human platelet-rich plasma samples were spiked with *E*. *coli* and six other bacterial species at 0.5–100 cfu/mL, following which the total DNA was coextracted for use in PCR analysis; five copies of the IRC were added to each reaction in the duplex PCR. Table 3 lists the data obtained from the 16S/23S IRC duplex PCR assays performed using the same conditions. The results show that *E*. *coli* detection was positive at 100, 10, and 5 cfu/mL but negative at 0.5 cfu/mL (*P* < 0.05). However, Cts and Cti were not significantly different (16S assays, 1 cfu/mL, *P* = 0.41; 23S assays, 1 cfu/mL, *P* = 0.12); thus, we performed a two-sample pooled *t* test (Materials and Methods) to test the hypothesis that Cts ≤ Cti. The *t*_*c*_ values calculated from the 16S and 23S assay results were −0.521 and 1.37, respectively, whereas *t*_*(0*.*10*, *6)*_ was 1.440; thus, *t*_*c*_ < *t*_*(0*.*10*, *6)*_, and we could not reject *H*_*0*_ at the 10% confidence level. Therefore, 1–2 cfu/mL of *E*. *coli* was designated as positive because the statistical analysis supported the hypothesis that Cts ≤ Cti. The data in Table 4 depict the performance of the IRC duplex RT-PCR assay for six bacterial species in spiked platelet-rich plasma samples; the results demonstrate that sensitivity was lower for *P*. *acnes*, *S*. *aureus*, and *S*. *epidermidis* than for the other species. In another experiment, we extracted bacterial gDNA, added it to 500 ng of human gDNA, and then performed the IRC duplex PCR analysis. The results showed that in this assay, one or two copies of these species could be detected (Table 4).

**Table 3:** Internal reference control (IRC) duplex RT-PCR testing of human platelet-rich plasma spiked with *E*. *coli*.

	Number of spiked*E*. *coli* (cfu/mL)	Mean Cts of target	Mean Cti of IRC	Positive/Negative
16S	100	26.72 ± 0.21	36.77 ± 0.12	Positive: *P* < 0.05
	10	29.42 ± 0.27	36.61 ± 0.24	Positive: *P* < 0.05
	5	32.39 ± 0.43	36.17 ± 0.51	Positive: *P* < 0.05
	1	36.31 ± 0.64	36.39 ± 0.27	Positive*: *P* = 0.41
	0.5	37.26 ± 0.33	36.30 ± 0.32	Negative: *P* < 0.05
23S	100	25.89 ± 0.21	35.82 ± 0.25	Positive: *P* < 0.05
	10	29.60 ± 0.40	36.41 ± 0.15	Positive: *P* < 0.05
	5	31.84 ± 0.28	35.64 ± 0.37	Positive: *P* < 0.05
	1	35.31 ± 0.18	35.14 ± 0.17	Positive*: *P* = 0.12
	0.5	36.64 ± 0.44	36.01 ± 0.26	Negative: *P* < 0.05

*The *P* value showed no difference between Cts and Cti; however, the two sample pooled *t*-test analysis showed that Cts ≤ Cti.

**Table 4:** Results of the spiking study in which the detectability of 16S/23S was tested using internal reference control (IRC) duplex RT-PCR.

Bacteria	Platelet-rich plasma spiked withbacterial culture (cfu/mL)		500 ng human gDNA spiked withbacterial gDNA (copies)	
	16S	23S	16S	23S
*B*. *cereus*	20	20	2	2
*E*. *cloacae*	10	10	2	1
*P*. *aeruginosa*	5	5	1	2
*P*. *acnes*	50	50	2	2
*S*. *aureus*	30	30	2	2
*S*. *epidermidis*	50	50	5	2

## Discussions

Diverse methods are available for detecting bacterial contamination [13]. RT-PCR-based techniques offer a potential alternative to conventional culture methods and these PCR methods could be used for detecting bacterial contamination as widely as for viral screening in transfusion medicine [14]. However, implementation of RT-PCR for universal bacterial contamination screening is hindered by certain factors, including the requirement for appropriate quality control, the choice of the target for detection, the bacterial DNA contamination of reagents, and the need for an ideal sampling strategy [15].

Previously, certain housekeeping genes such as human mitochondrial DNA (mtDNA) genes [12], human beta-microglobulin mRNA [16], and the HLA-DQA gene [17] were used as internal controls to monitor the efficacy of DNA coextraction and subsequent amplification. However, one disadvantage of using these controls is that the assay results might not accurately reflect target amplification; this is because the use of distinct primer sets inevitably leads to competition between the primers. Rood et al. [8] demonstrated that recombinant lambda phage could be used as the internal control: the phage contained an artificial DNA fragment that shared the same primer set with the 16S target [8]. In comparison, the IRC duplex RT-PCR format reported herein offers certain advantages: (1) the IRC used here can be produced and standardized more easily than the lambda phage internal control, (2) the precise copy number of the IRC molecules can be readily manipulated and the minimum copy number for the positive detection of the internal control or other quantity reference controls can be set, (3) the IRC can be used for preparing the standard curve, and (4) a two-sample pooled *t*-test statistical method is incorporated into the data analysis in order to establish true positive results and enhance reliability. When performing RT-PCR-based assays for universal bacterial screening, choosing the target for detection can be highly challenging because representative reference standards for distinct bacteria are not available. Consequently, fragments of conserved genes (e.g., 16S/23S rRNA, *tuf*, *rpoB*, and *groEL*) have been shown to be well-suited for broad-range bacterial detection at approximately the same efficiency and specificity [7,16,17]. Here, we selected 16S and 23S rRNA genes as candidates for redesigning the primers and probes. Our results indicate that regardless of the type of target gene selected, the IRC duplex RT-PCR format can potentially serve as a favorable method of nucleic acid detection.

The contamination of reagents and consumables with traces of exogenous bacterial DNA represents an intractable problem, particularly in the case of PCR-based universal bacterial detection. Commonly, nonspecific exogenous DNA is digested using DNase I or restriction endonucleases (e.g., *Mse*I, *Sau*3AI) [9,18]. Another promising method involves using a photo-activated DNA-eliminating agent such as 8-methoxypsoralen or ethidium monoazide [7,12]. However, most of these decontamination methods are not entirely effective [19], and they can affect PCR sensitivity or amplification efficiency. In this study, the lower limits of detection were determined for *P*. *acnes*, *S*. *aureus*, and *S*. *epidermidis*. We speculate that this might have resulted from the inefficiency of the DNA preparation for these microorganisms (particularly gram-positive bacteria) in comparison to other microorganisms. Thus, we used alternative DNA isolation procedures, which included the use of distinct blood DNA isolation kits; however, these kits failed to isolate a minor amount of bacterial gDNA for certain gram-positive bacteria and bacteria possessing a very thick cell walls (data no shown). This partly supported the notion that the method used to process samples might affect the sensitivity of the assay [15].

## Conclusions

We sought to develop an assay format in which an IRC and quantity control are included to improve the efficiency and reliability of RT-PCR-based methods for detecting bacterial contamination in blood components. The designed synthetic IRC can be used for minimizing false-negative results, and the statistical determination of whether Cts ≤ Cti can ensure that the detected target is a true positive. The IRC duplex RT-PCR assay will be useful for infectious risk screening in the field of blood-supply safety, wherein false-negative and false-positive results must be limited and the authenticity of positive results must be established.
